# A comparison of Gam-COVID-Vac vaccination and non-vaccination on neurological symptoms and immune response in post-COVID-19 syndrome

**DOI:** 10.5339/qmj.2025.6

**Published:** 2025-02-23

**Authors:** Saulesh Kurmangaliyeva, Akzhan Madenbayeva, Saltanat Urazayeva, Kristina Baktikulova, Kairat Kurmangaliyev

**Affiliations:** ^1^Department of Microbiology, Virology and Immunology, West Kazakhstan Marat Ospanov Medical University, Aktobe, Republic of Kazakhstan; ^2^Department of Epidemiology, West Kazakhstan Marat Ospanov Medical University, Aktobe, Republic of Kazakhstan; ^3^Department of Transfusiology, West Kazakhstan Marat Ospanov Medical University, Aktobe, Republic of Kazakhstan *Correspondence: Saltanat Urazayeva. Email: sal.urazayeva@gmail.com

**Keywords:** Post-COVID-19, long COVID-19, coronavirus, cellular and humoral immunity, hybrid immunity, damage to the nervous system

## Abstract

The post-COVID-19 syndrome may present with a range of neurological symptoms such as headaches, sleep disorders, and dizziness. The objective of this study was to examine the effectiveness of the Gam-COVID-Vac vaccine in mitigating the neurological symptoms of post-COVID-19 syndrome. The study involved 95 patients diagnosed with the neurological form of long COVID-19, who were divided into two groups according to their vaccination status. The immunological parameters of humoral immunity were evaluated by enzyme-linked immunosorbent assay (ELISA), while the parameters of cellular immunity were evaluated using flow cytometry. Administration of the vaccination resulted in a reduction in clinical symptoms of the neurological form of long COVID-19. Statistically significant differences (*p* = 0.035) were found in symptoms such as headaches, sleep disturbances, and dizziness, especially in central nervous system (CNS) disorders, between the groups that received the vaccination and those that did not. More than 90% of patients had elevated levels of Receptor Binding Domain (RBD) immunoglobulin G against the viral S-protein (>2,500 BAU/ml), indicating strong humoral immunity regardless of vaccination status. An increase in B-lymphocyte (CD3^-^CD19^+^) counts was noted in both groups, with levels significantly higher in the group that received the vaccination (*p* < 0.03). Analysis of T-cell profiles and NK (natural killer) cell levels showed no changes. The study suggests that administration of Gam-COVID-Vac vaccination could reduce the occurrence of CNS symptoms in individuals with post-COVID-19 syndrome. Although certain neurological symptoms may continue, immunization has a beneficial influence on their progression. The results emphasize the crucial role of an increased humoral immune response in individuals with post-COVID-19 syndrome, but do not show significant changes in T-cell immune parameters.

## INTRODUCTION

Post-COVID-19 potentially affects almost any system of the body, leading to respiratory, neurological, psychiatric, cardiovascular, and gastrointestinal disorders. Recently, attention has focused on potential neurological consequences that may manifest after the onset of COVID-19 symptoms.^1^ The clinical outcomes of SARS-CoV-2 remain unclear, as does the possibility of progression of neurological disorders. Neurological complications of COVID-19 were first described by Mao et al.,^2^ including central nervous system (CNS) disorders such as epilepsy, stroke, headaches, altered consciousness, ataxia, dizziness, as well as peripheral nervous system disorders such as taste and smell disorders, neuropathies, visual impairments, and neuralgia.

Neurological signs are often the main symptoms of coronavirus infection.^3^ They may also be one of the variants of post-COVID-19 syndrome. Studying the neurovirulence of COVID-19 is a complex task. Research has been conducted on the effect of coronavirus virions on the CNS. Viral ribonucleic acid is often present in the cerebrospinal fluid of patients with signs of meningitis and encephalitis, as well as in postmortem brain samples for infection identification.^4-6^ Virus penetration into neurons has been documented in model objects (cell cultures and animals).^7^ These studies demonstrate the neurotropic potential of the virus. Two pathways are presumed for virus penetration into the nervous system: overcoming the blood–brain barrier or penetration through the olfactory bulb and cribriform plate.^8,9^

In many cases, mild forms of the disease occur, accompanied by an active immune response. In some patients, a severe form develops with weak activation of immune links.^10,11^ Both humoral and cellular mechanisms of the body's response to the coronavirus are well studied.^12–14^ Although elevated immunoglobulin G (IgG) levels in serum may correlate with the intensity of most symptoms, no differences in T-cell responsiveness were observed for most infection symptoms. It has been proven that coughing and reduced sensitivity to taste and smell are associated with a decrease in the intensity of the T-cell response. However, the association of T-cell immunity with long COVID-19 variants has not yet been investigated. T cells not only play a central role in the immune activation of antibody-producing clones, but also facilitate virus clearance through direct lysis of infected cells.^15,16^

Several individual reports and case series suggest that post-COVID-19 syndrome symptoms may be alleviated after vaccination.^17^ There are also several studies that suggest both symptom relief and exacerbation after vaccination in previously infected patients.^18,19^ In the Republic of Kazakhstan, mass vaccination against COVID-19 began in February 2021. One of the vaccines used was Gam-COVID-Vac. The vaccine is based on the adenovirus vectors Ad5 and Ad26. They deliver the complex S-protein gene of the coronavirus to the target, but cannot “reproduce” in the body. Such a vaccine induces high antibody titers and a comprehensive immune response.

The objective of this study was to assess the effectiveness of the Gam-COVID-Vac vaccine in mitigating the neurological symptoms of post-COVID-19 syndrome, particularly those associated with the CNS. The researchers also examined immunological parameters such as antibody levels and different subtypes of immune cells to gain a deeper understanding of immune response mechanisms in patients with the neurological form of post-COVID-19 syndrome. To evaluate the long-term effect of vaccination on neurological complications of COVID-19, the study aimed to identify differences in clinical manifestations and immunological profiles between vaccinated and unvaccinated participants one year after acute infection or vaccination.

## SUBJECTS AND METHODS

The study involved 95 patients with post-COVID-19 syndrome. The age range of the participants was 18–72 years. Depending on the vaccination status, two groups were formed: group I, consisting of 49 patients with a history of COVID-19 who received the Gam-COVID-Vac vaccine, and group II, consisting of 46 patients with a history of COVID-19 who were not vaccinated. The study did not include children and pregnant women, lactating women, individuals over 75 years old, or individuals with severe comorbidities (oncological diseases of any localization, autoimmune diseases, primary and secondary immunodeficiencies). No complex side effects were observed after vaccination. The study was not a clinical trial, and there was no interference with the vaccination of participants. Instead, the study reviewed medical records and conducted an observational study with 95 participants one year after vaccination or infection.

Study conditions complied with the World Medical Association Declaration of Helsinki – Ethical Principles for Medical Research Involving Human Subjects. All individuals participating in the study signed informed consent forms. The informed consent form was reviewed and approved by the local ethics committee of Marat Ospanov West Kazakhstan Medical University (no. 7.07, dated 28 September 2022). Information registration cards were filled out for all patients to assess clinical and laboratory parameters. The diagnosis of post-COVID-19 syndrome was made according to the protocol for the diagnosis and treatment of complications after coronavirus infection (post-COVID-19 syndrome) in adults.^20^

To examine immunological parameters in both groups with post-COVID-19 syndrome, fasting peripheral blood analysis was conducted. In group 1 (vaccinated), indicators of cellular and humoral immunity were determined one year after the full course of vaccination, while in group 2, indicators of cellular and humoral immunity were determined one year after acute COVID-19 infection. The humoral response was evaluated using the enzyme-linked immunosorbent assay (ELISA) method to determine the presence of neutralizing IgG against the receptor-specific region RBD of S-glycoprotein of coronavirus particles. The “SARS-CoV-2 IgG ELISA Kit” from Euroimmun was used as the ELISA kit.

To examine cellular immunity, blood with the anticoagulant ethylenediaminetetraacetic acid was analyzed by flow cytometry. First, the number of granulocytes was determined, followed by each cell group separately, according to marker molecules: CD19^+^, CD3^+^HLA-DR, CD3^+^, CD3^+^CD16^+^CD56^+^, D3^+^CD8^+^, D3^-^CD16^+^CD56^+^, D3^+^CD4^+^. Phenotyping of peripheral blood lymphocytes was performed with the Beckman Coulter Navios flow cytometer using monoclonal antibodies (mAb) at the Marat Ospanov West Kazakhstan Medical University. Exclusion of hematocytes not relevant to the study was performed using mAb to CD45-labeled PerCP. The Beckman Coulter Navios flow cytometer and Kaluza software were used for the study.

Statistical analysis was performed using IBM SPSS Statistics software for Windows version 25. The arithmetic mean value and standard deviation [Me (SD)] were taken into account. Median, lower, and upper quartiles [Me (Q1; Q3)] were used in non-parametric statistics. The Shapiro–Wilk and Kolmogorov–Smirnov tests were used to characterize the normal and non-normal distribution of the data. Because the data were not normally distributed, non-parametric statistical methods were used. The Pearson chi-square test χ^2^ was calculated to compare the frequencies of qualitative variables and determine the odds ratio (OR) and 95% confidence interval (CI). Quantitative variables were compared based on the Mann–Whitney U-test. The difference was considered statistically significant if *p* ≤ 0.05.

## RESULTS

The diagnosis of COVID-19 was made on the basis of positive PCR results in the medical history, as well as a positive antibody test for the coronavirus.^21,22^ Overall, among the total number (95) of patients with post-COVID-19 condition, 46 (48.4%) were unvaccinated and 49 (51.6%) had received two doses of the Gam-COVID-Vac vaccine. The analysis of the examined parameters was conducted 12 months after the second vaccination.

As indicated in Table 1, among patients regardless of vaccination status, women were more common (*n* = 37 out of 49, 75.5% in the vaccinated group, and *n* = 38 out of 46, 82.6% in the unvaccinated group). Comparison of age groups between vaccinated and unvaccinated participants showed no statistically significant differences (*p* = 0.053), indicating that there was no effect of age on the distribution between the groups. Depending on the age group, individuals aged 41–60 years predominated among the unvaccinated (*n* = 23 out of 46; 50%), while younger patients aged 18–40 years were more prevalent in the vaccinated group (*n* = 27 out of 49; 55.1%). The smoking indicator showed statistically insignificant differences in both groups (*p* = 0.96). The nature of complications in patients did not depend on vaccination, and there was no statistically significant difference.

**Figure 1.**
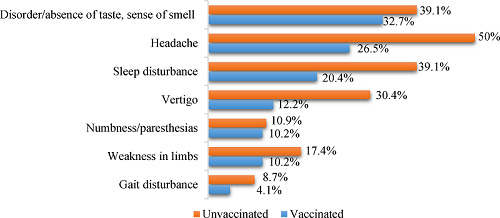
Symptom rates in cohorts of patients with post-COVID-19 syndrome.

When comparing the frequency of occurrence of the neurological variant of the post-COVID-19 syndrome between unvaccinated and vaccinated individuals, there were statistically significant differences (*p* = 0.035). The most common symptoms were disturbances in taste and smell, followed by headaches and sleep disorders, with a higher prevalence among unvaccinated patients (50% and 39.1%, respectively) (Figure 1). When clinical symptoms were compared, no association was observed between disease severity and gender, nor was there a correlation between the severity of the initial infection. Fewer complaints about indicators of peripheral nervous system involvement such as numbness, paresthesia, and gait disturbance were reported.

When comparing the frequency of occurrence of individual symptoms, statistically significant differences were found: headache OR 2.8, 95% CI 1.2–6.64, *p* = 0.018; sleep disturbance OR 2.6, 95% CI 1–6.24, *p* = 0.045; dizziness OR 3.21, 95% CI 1.20–8.95, *p* = 0.03. Symptoms such as numbness/paresthesia (OR 1.07, 95% CI 0.29–3.98, *p* = 0.916), limb weakness (OR 1.85, 95% CI 0.56–6.14, *p* = 0.308), gait disturbance (OR 2.238, 95% CI 0.42–12.849, *p* = 0.356), and impaired taste and smell (OR 1.32, 95% CI 0.572–3.07, *p* = 0.51) showed statistically insignificant differences (Table 2).

All participants suffered from neurological symptoms following coronavirus infection one year after illness, but the unvaccinated reported more frequent manifestations (35.7% vs. 27.3%, *p* = 0.035) (Table 3).

Table 3 shows the average IgG titers against the S-protein of the coronavirus in the serum of participants with post-coronavirus symptoms. Antibody titers were compared between the two groups and no significant differences were found between vaccinated and unvaccinated individuals with long-term COVID-19. The results of comparing RBD IgG levels with the SARS-CoV-2 S-glycoprotein in vaccinated and unvaccinated samples showed statistically insignificant differences (*p* = 0.547). More than 90% of patients with post-COVID-19 syndrome, predominantly with a neurological variant, had RBD IgG levels against the SARS-CoV-2 S-protein >2,500 BAU/ml. High antibody titers were found in individuals regardless of the severity of the disease.

The status of specific humoral immunity against the coronavirus in cases of post-COVID-19 syndrome plays a key role in understanding the immunogenesis of the disease against the backdrop of different vaccination statuses.^23^ A more significant role in the response to coronavirus infection is attributed to the humoral response because this mechanism is mainly used to construct vaccination vectors, and immunoglobulins can be conveniently determined in numerical samples. Nevertheless, patients have strong humoral immunity against prolonged COVID-19 regardless of vaccination status. There were no statistically significant differences in IgG titers against the S-glycoprotein of the coronavirus between vaccinated and unvaccinated patients of different genders and age groups.^21,22^ For this reason, gender and age criteria were not highlighted in the study.

Both humoral and cellular cascades play an important role in post-vaccination and post-infection immune responses. In particular, cellular immunity provides a significant level of antiviral protection.^24^ The next task was to determine the different types of cell subpopulations and ascertain which cell types correlate with protective immunity in individuals with post-coronavirus symptoms. The study of cellular immunity in patients suffering from long-term COVID-19 infection focuses on parameters such as T and B cell subpopulations to assess their changes in the post-COVID-19 context and post-COVID-19 vaccination. Immune parameters were analyzed in 95 patients with post-COVID-19 syndrome who presented neurological symptoms (49 vaccinated and 46 unvaccinated against SARS-CoV-2) (Table 4).

In summary, when comparing immune parameters in both groups, there was an increased number of B-lymphocytes (CD3^-^CD19^+^), a reduced number of natural killer (NK) cell subpopulations (CD3^+^CD56^+^CD16^+^), and a slight increase in the immunoregulatory index (CD4/CD8) in the unvaccinated group compared to vaccinated patients. However, other panels of cellular immunity in both comparison groups remained within normal ranges.

Overall, the T-cell parameters did not exceed the reference range in all participants. In the comparative analysis, the CD3^+^ indicator remained within the reference values, but there was a statistically significant increase in CD3^+^ in the vaccinated patient group (*p* = 0.01). To date, numerous data show that the T-lymphocyte response is important both for the initial cessation of viral particle replication and for monitoring the condition during the course of the infectious process.^25^ It is well known that T-lymphocytes are crucial for the formation of high-affinity antibodies and immune memory, as they activate the cascade of differentiation and maturation of B-lymphocytes. Changes in the immune profile, such as increased levels of B-lymphocytes (CD3^-^CD19^+^), were noted in both cohorts during the research, with significantly more CD3^-^CD19^+^ cells present in the vaccinated cohort compared to the unvaccinated cohort (*p* < 0.03), indicating the development of a robust hybrid humoral response and the synthesis of specific immunoglobulins. Given the profound and prolonged dysregulation of the immune response in both acute disease and post-COVID-19, there may be a compensatory increase in B-lymphocytes and increased antibody production in individuals with post-coronavirus syndrome.

**Figure 2.**
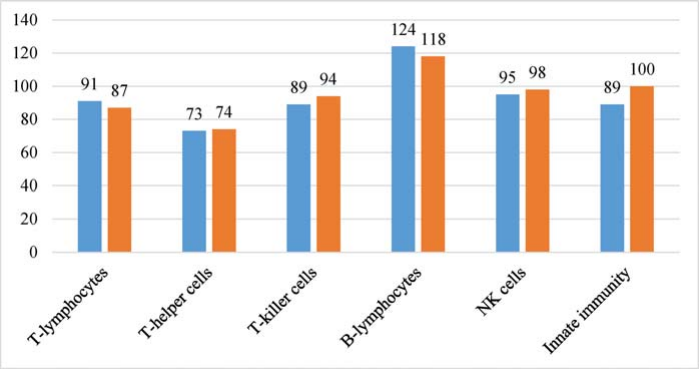
Immunological parameters of patients in percentage equivalent relative to the average reference value. Blue: vaccinated patients, orange: unvaccinated patients, NK cells: natural killer cells, IRI: immunoregulatory index.

In the study, there was no decrease in the levels of innate immune defense factors: NK cells (CD3^-^CD16^+^56^+^) remained within the reference values. A decrease in the relative content of NK T cells (CD3^+^CD56^+^CD16^+^) was observed. Regarding helper T cells (CD3^+^CD4^+^), cytotoxic T cells (CD3^+^CD8^+^), and double-positive T-lymphocytes (CD4^+^CD8^+^), no significant deviations from reference values were found in both comparison groups, and no statistical differences were found between the investigated groups. The titers of immune cells with different markers (in percentage equivalent) relative to the commonly accepted average reference values are shown in Figure 2.

As shown in Figure 2, all indicators in both groups, except the titer of B-lymphocytes, are below the lower limit of the mean reference value. Vaccinated patients have 5% more T cells compared to unvaccinated patients. However, vaccinated individuals have 5% fewer killer T cells, 3% fewer NK cells, and an 11% lower immunoregulatory index. The difference in the number of helper T cells between the samples is within 1%. The titer of B-lymphocytes is above the mean reference value in both groups, and 6% higher in the vaccinated sample than the corresponding value in the unvaccinated sample.

A comparison was also made between the severity of COVID-19 symptoms and the specific response of T-lymphocytes to monocyte epitopes restricted by HLA class II, HLA-DR. The intensity of T-lymphocyte responses to HLA-DR did not differ for individual symptoms or between the two samples (*p* = 0.82).

The immunoregulatory index indicates the ratio of helper T-lymphocytes to suppressor T cells: the higher it is, the more helpers and the fewer suppressors. Presumably, the index is lower in the vaccinated group than in the unvaccinated group because the vaccine response is accompanied by the overproduction of antibodies, and there is a sequential partial inhibition of immune activity after the accumulation of immunoglobulins.

A significant decrease in CNS symptoms, including headaches, sleep disturbances, and dizziness, was observed in participants who were administered the Gam-COVID-Vac vaccine compared to those who were not vaccinated. In the immunized group, the likelihood of developing neurological symptoms was reduced by 2.5 times. Approximately 27% of those vaccinated reported persistent neurological symptoms in the year following infection, with headaches and dizziness being the most prevalent. Unvaccinated individuals showed a higher incidence and intensity of CNS symptoms, with 36% of them experiencing persistent neurological symptoms. These symptoms included headaches, sleep disorders, vertigo, and changes in taste and smell. Higher rates of headaches (50%) and dizziness (39.1%) were observed in the unvaccinated group, indicating a greater likelihood of experiencing CNS-related symptoms.

A strong humoral immune response was found in the vaccinated group, as more than 90% of participants had increased levels of RBD-specific IgG antibodies (>2,500 BAU/ml) against the S-protein of SARS-CoV-2. Furthermore, the group that received the vaccination had significantly elevated levels of B-lymphocytes (CD3^-^CD19^+^) compared to the group that did not receive the vaccination (*p* < 0.03). Nevertheless, T cell and NK cell levels remained within the usual range and showed no significant differences between vaccinated and unvaccinated individuals. The unvaccinated group showed robust humoral immunity, as evidenced by over 90% of members having increased levels of IgG antibodies, which was comparable to the vaccinated group. Although B-lymphocyte levels were increased, they were significantly reduced compared to the vaccinated group. There were no significant changes in T-cell profiles or NK cell levels following vaccination.

Therefore, the number and severity of neurological symptoms experienced by vaccinated individuals were lower than those who were naturally infected with COVID-19, particularly CNS dysfunction. Both groups showed robust humoral immune responses, but vaccine recipients had elevated B-lymphocyte levels, suggesting stronger immune response in the vaccinated group. Neither group showed significant changes in T-cell or NK cell counts. In general, immunization was associated with decreased neurological complaints and increased humoral immune responses.

## DISCUSSION

The study was conducted approximately one year after COVID-19 in the unvaccinated participants and after vaccination in the vaccinated group. Regardless of the passage of time, a significant number of participants in both groups continued to experience neurological symptoms such as headaches, sleep disturbances, dizziness, and taste and smell disorders. This suggests that certain neurological manifestations of COVID-19 may persist and endure beyond the initial phase of the illness. Nevertheless, there was a significant reduction in symptoms related to CNS impairment (such as headaches, sleep disturbances, and dizziness) in individuals who received the vaccine compared to those who did not receive the vaccine. The unvaccinated participants were 2.5 times more likely to develop neurological symptoms. No significant differences were observed between groups in terms of symptoms associated with the peripheral nervous system, such as numbness/paresthesias, limb weakness, and gait disturbance. Although certain neurological symptoms may persist for an extended period of time, vaccination has a positive impact on the course of CNS symptom development. However, further monitoring is required to fully evaluate the long-term consequences.

Among vaccinated patients, headaches were reported in 27% of individuals and dizziness was reported in 12%, which is consistent with data from other researchers: approximately 25% of patients suffered from headaches and 9% experienced dizziness.^26–29^ According to Zhou et al.,^30^ neurological symptoms during COVID-19 infection are due to the penetration of viral particles through the blood–brain barrier due to viremia or direct entry into neurons through the olfactory bulbs. Subsequently, the virus binds to specific receptors, penetrates neurons, and induces their death. The immunological and epidemiological effectiveness of the Gam-COVID-Vac vaccine is illustrated by its prolonged use during the pandemic. Several studies have been conducted on plasma samples from individuals who had COVID-19 and were then vaccinated with the Gam-COVID-Vac vaccine, showing robust post-vaccination immunity even after a single dose. Moreover, no dependence of the post-vaccination immune response on the age and gender of the participants could be observed.^31^

According to Uysal et al.,^32^ after the re-administration of CoronaVac to different recipient groups, the antibody titer varied over different time ranges after the second dose. On average, the antibody level in the study conducted was approximately 100 BAU/ml. However, accompanying factors did not affect the antibody titer in the present study. The amount of antibodies after vaccination depended on the participants’ body mass index, smoking or non-smoking status, and age. It is important to emphasize that the recipients did not previously have a coronavirus infection. Antibody levels were observed to be different among different age groups. For example, individuals aged 40–49 years showed a decrease in antibody levels, while those aged 20–29 years showed an increase. The highest antibody levels were found in the age group of 30–39 years.

In the initial stage of infection, the immune response to the virus occurs in two ways: cellular and humoral. The cellular response is mediated by T-lymphocytes, which recognize viral antigens bound to class I and II histocompatibility complexes and subsequently neutralize infected cells. The humoral response begins with the synthesis of multivalent antiviral IgM by “unspecialized” B-lymphocytes. Immunophenotypic memory B and T cells are subsequently formed, which can persist for several months. The latter shows reactivity against several epitopes of SARS-CoV-2.^33^ The functional aspect of hybrid and natural immunity in the post-coronavirus period (including past infections and vaccinations) has not been fully elucidated.^25,34^ There is evidence of a decrease in the number of NK cells and a simultaneous increase in the titer of B-lymphocytes and T cells, especially helper T cells and TNK lymphocytes, in post-COVID-19 studies.^35,36^

In the present study, an increase in the number of B-lymphocytes and a simultaneous partial decrease in the NK titer were also observed in patients, regardless of vaccination status. Moreover, there was a 6% increase in CD19^+^ marker cells in the vaccinated group compared to the unvaccinated group, while CD16^+^CD56^+^ cells were slightly lower. The antibody titer remained high in both groups because they had a history of coronavirus infection. However, patients with neurological disorders who did not have the coronavirus showed a stimulation of the humoral response to the virus after receiving a vaccine and booster.^37,38^ It was shown that there was a rapid increase in T cell titer and the concentration of gamma-interferon after the second vaccination in the study group. The latter parameter strongly correlated with the synthesis of IL-2 and tumor necrosis factor-alpha, confirming a coordinated T-cell response to vaccination. Over time, the antibody titer partially decreased to 33 BAU/ml after the second injection and slightly increased after the booster vaccination (five months after the second dose). The booster injection also slightly increased the number of T-lymphocytes, the titer of which decreased between the second and third doses. These data demonstrate the effectiveness of the vaccination in patients with neurological disorders who have not yet contracted the infection.

The impact of the course of a coronavirus infection in unvaccinated individuals on IgG levels and immune B cells remains unclear. As indicated by Kato et al.,^39^ the concentration of IgG remained proportional up to 8 weeks after the diagnosis of coronavirus infection in two groups with different (asymptomatic and symptomatic) variants of the infectious process. In the present study, no changes in antibody titer were observed depending on the type of symptomatic complication. In the control sample of the researchers, the titer of specific memory cells capable of producing coronavirus antibodies was limited to 20 × 10^6^ cells per ml. Recovering individuals showed a 75% increase in B-lymphocyte levels. In this case, the symptomatic and asymptomatic variants did not differ in this parameter.

Current and preliminary studies demonstrate an active and regulated comprehensive immune response to the coronavirus in both people with neurological disorders and those without accompanying pathologies. Timely vaccination helps strengthen both cellular and humoral immunity.^40^

To ensure the strength and limitations of future studies on the impact of vaccination on post-COVID-19 neurological symptoms, it is imperative to include several essential components. Firstly, the focus was not on past infections, although this factor influences the severity of the disease course after COVID-19. Secondly, performing subgroup analysis that takes into account demographic variables and disease severity will provide deeper insights into the different responses to vaccination and the occurrence of neurological symptoms after COVID-19. Inclusion of a longitudinal follow-up component is crucial to assess the evolution of these symptoms over time and to determine whether vaccination affects the emergence of neurological disorders. This methodology would facilitate the detection of any delayed or inconspicuous impacts of vaccination on neurological results and provide valuable information about the lasting protection that vaccines provide against post-COVID-19 complications. Furthermore, it is important to perform regular evaluations of immune parameters at different intervals during the follow-up period to understand the changes in vaccine-induced immune responses over time. This involves tracking changes in antibody titers, T-cell profiles, and other immune markers beyond the first year, thereby providing information about long-lasting and consistent vaccine-induced immunity against neurological complications resulting from COVID-19. Incorporating patient-reported outcomes, such as measures of quality of life or scales assessing functional disability, will enable a more comprehensive assessment of the broader effects of post-COVID-19 neurological symptoms on individuals. To improve the reliability of the study and obtain more dependable evidence on the impact of vaccination on post-COVID-19 neurological complications, it is recommended to increase the sample size and ensure a wider range of participants in terms of diversity. The enhancements will greatly improve the study design and contribute to a more comprehensive understanding of the complex interplay between COVID-19, vaccination, and neurological well-being.

## CONCLUSIONS

During the study, it was found that receiving the COVID-19 vaccine is not associated with worsening symptoms of the post-COVID-19 neurological variant. Vaccinated individuals showed a reduction in symptoms of infection, especially of the CNS: neurological symptoms were 2.5 times more common in the unvaccinated group. Complications such as headaches, sleep disturbances, and dizziness occurred significantly less frequently. One year after vaccination, 27% of the individuals were diagnosed with neurological symptoms, compared to 36% of unvaccinated individuals. These results confirmed the effectiveness of coronavirus vaccination regardless of infection and indicated the presence of intense natural and hybrid humoral immunity in patients with the post-COVID-19 neurological variant.

During the study, intense humoral immunity was found associated with prolonged COVID-19 symptoms, regardless of vaccination status. More than 90% of patients with post-COVID-19 syndrome, predominantly the neurological variant, had a high titer of IgG antibodies against the S-protein (above 2,500 BAU/ml). No significant changes in T-cell parameters were found, but an increase in B-lymphocytes (CD3^-^CD19^+^) in both groups, with the CD3^-^CD19^+^ subset being significantly higher in the vaccinated sample, as well as a decrease in the relative content of NK cell subpopulations. The B-lymphocyte titer exceeded the mean reference value by 24% in the vaccinated sample and by 18% in the unvaccinated sample. The numbers of helper T cells, killer T cells, and NK cells in both vaccinated and unvaccinated patients did not exceed the reference value, but were below the mean value. At the same time, an increase in the immunoregulatory index was observed in the unvaccinated sample.

Further observation of patients with the neurological variant of post-COVID-19 and analysis of their immunograms are necessary because the course of post-COVID-19 syndrome is unpredictable and heterogeneous, varying between different individuals depending on various factors.

### Funding

This study was carried out within the framework of the scientific project “The effect of vaccination on immunity in patients with the condition after COVID-19 in the population of Kazakhstan” (IRN AP14870878) for the period 2022–2024 with grant funding from the Science Committee of the Ministry of Science and Higher Education of the Republic of Kazakhstan.

### Conflict of interest

The authors have no conflicts of interest to declare.

## Figures and Tables

**Table 1.** Sociodemographic and medical history of participants with post-coronavirus syndrome according to vaccination status.

	**Vaccinated (*n*=49)**	**Not vaccinated (*n*=46)**	***p***

	**Gender, *n* (%)**		0.396

Female	37 (75.5)	38 (82.6)	

Male	12 (24.5)	8 (17.4)	

	**Age groups (years), *n* (%)**		0.053

18–40	27 (55.1)	14 (30.4)	

41–60	16 (32.7)	23 (50)	

>60	6 (12.2)	9 (19.6)	

	**Smoking, *n* (%)**		0.936

Smoker	3/49 (6.1)	3/46 (6.5)	

Non-smoker	46/49 (93.9)	43/46 (93.5)	

	**BMI, *n* (%)**		0.058

Underweight	2/49 (4.1)	0/46 (0)	

Normal weight	30/49 (61.2)	21/46 (45.7)	

Overweight	13/49 (26.5)	13/46(28.3)	

Obesity	4/49 (8.2)	12/46 (26.1)	

	**Comorbidity, *n* (%)**		0.211

0	32/49 (65.3)	27/46 (58.7)	

1	13/49 (26.5)	12/46 (26.1)	

2	0/49 (0)	4/46 (8.7)	

3	4/49 (8.2)	3/46 (6.5)	

	**Comorbidity, *n* (%)**		

Arterial hypertension	9/49 (18.4)	12/46 (26.1)	0.365

Obesity	4/49 (8.2)	12/46 (26.1)	0.058

Diabetes	4/49 (8.2)	6/46 (13)	0.439

Cardiovascular diseases	1/49 (2)	2/46 (4.3)	0.520

Chronic lung diseases	5/49 (10.2)	4/46 (8.7)	0.802


**Table 2.** Odds ratio (OR) of neurological symptoms in vaccinated and unvaccinated participants.

**Symptoms**	**OR**	***p***

Headache	OR 2.8, 95% CI 1.2–6.64	0.018

Sleep disturbance	OR 2.6, 95% CI 1–6.24	0.045

Numbness/paresthesia	OR 1.07, 95% CI 0.29–3.98	0.916

Limb weakness	OR 1.85, 95% CI 0.56–6.14	0.308

Gait disturbance	OR 2.238, 95% CI 0.42–12.849	0.356

Impaired taste and smell	OR 1.32, 95% CI 0.572–3.07	0.51

Dizziness	OR 3.21, 95% CI 1.2–8.95	0.03

Neurological variant	OR 2.506, 95% CI 1.056–5.952	0.035


**Table 3.** Coronavirus S-protein IgG levels in participants with post-coronavirus syndrome according to vaccination status.

**RBD IgG to coronavirus S-glycoprotein (BAU/ml)**	**Vaccinated**	**Unvaccinated**	***p***

< 7.1	1/49 (2%)	0/46 (0%)	

7.1–2,500	2/49 (4.1%)	3/46 (6.5%)	0.547

>2,500	46/49 (93.9%)	43/46 (93.5%)	


**Table 4.** Immunogram parameters of vaccinated and unvaccinated participants with post-coronavirus symptoms [Me (Q1; Q3)].

**Options**	**Vaccinated (*n*=49)**	**Not vaccinated (*n*=46)**	***p***

CD3	70 (67.8; 74.2)	67.2 (63.4; 72.3)	0.01

CD3^-^CD19^+^	14.8 (12.4; 16.3)	14 (11.2; 16)	0.03

CD3^+^CD4^+^	34.4 (26.8; 37.7)	35 (28.4; 38)	0.7

CD3^+^CD8^+^	24.6 (22; 28.2)	26 (21.8; 28.4)	0.59

CD4^+^CD8^+^	0.7 (0.18; 2)	0.4 (0.18; 2.2)	0.88

CD4/CD8	1.6 (1.2; 1.8)	1.8 (1.5; 2)	0.06

CD3^+^CD16^+^56^+^	4.4 (3.5; 6.9)	4.55 (3; 6.9)	0.79

CD3^-^CD16^+^56^+^	9.4 (6.75; 12.8)	9.7 (6.8; 12.4)	0.75

CD3^+^HLA^-^DR^+^	5.6 (4; 10)	6.9 (4; 12)	0.19

HLA^-^DR^+^	12.05 (6.8; 16.4)	12.3 (4.3; 17.8)	0.82

CD3^+^CD25^+^	2.5 (0.4; 3.2)	2.9 (0.5; 4)	0.28

